# Diagnostic Accuracy of Whole-Body Computed Tomography for Incidental Ovarian Tumors in Patients with Prior Breast Cancer

**DOI:** 10.3390/diagnostics12020347

**Published:** 2022-01-29

**Authors:** Pei-Ching Huang, Ren-Chin Wu, Yu-Hsiang Juan, Hui-Yu Ho, Yung-Chang Lin, Yi-Ting Huang, Shu-Hang Ng, Chyong-Huey Lai, Angel Chao, Gigin Lin

**Affiliations:** 1 Department of Medical Imaging and Intervention, Chang Gung Memorial Hospital at Linkou, No. 5, Fuxing St., Guishan Dist., Taoyuan City 33382, Taiwan; spookie@cgmh.org.tw (P.-C.H.); jonat126@yahoo.com.tw (Y.-H.J.); shuhangng@gmail.com (S.-H.N.); 2 Department of Medical Imaging and Radiological Sciences, Chang Gung University, No. 259, Wenhua 1st Rd., Guishan Dist., Taoyuan City 33302, Taiwan; hyt3784@cgmh.org.tw; 3 Department of Pathology, Chang Gung Memorial Hospital at Linkou, No. 5, Fuxing St., Guishan Dist., Taoyuan City 33382, Taiwan; renchin.wu@gmail.com; 4 School of Medicine, College of Medicine, Chang Gung University, No. 259, Wenhua 1st Rd., Guishan Dist., Taoyuan City 33302, Taiwan; brook.ho0229@gmail.com (H.-Y.H.); yclinof@adm.cgmh.org.tw (Y.-C.L.); laich46@cgmh.org.tw (C.-H.L.); Drangiechao@gmail.com (A.C.); 5 Department of General Surgery, Chang Gung Memorial Hospital at Linkou, No. 5, Fuxing St., Guishan Dist., Taoyuan City 33382, Taiwan; 6 Clinical Metabolomics Core Lab, Chang Gung Memorial Hospital at Linkou, No. 5, Fuxing St., Guishan Dist., Taoyuan City 33382, Taiwan; 7 Division of Medical Oncology, Department of Internal Medicine, Chang Gung Memorial Hospital at Linkou, No. 5, Fuxing St., Guishan Dist., Taoyuan City 33382, Taiwan; 8 Department of Radiology Oncology, Chang Gung Memorial Hospital at Linkou, No. 5, Fuxing St., Guishan Dist., Taoyuan City 33382, Taiwan; 9 Department of Obstetrics and Gynecology and Gynecologic, Cancer Research Center, Chang Gung Memorial Hospital at Linkou, No. 5, Fuxing St., Guishan Dist., Taoyuan City 33382, Taiwan

**Keywords:** breast neoplasms, computed tomography, metastasis, ovarian neoplasms

## Abstract

Whole-body computed tomography (WBCT) serves as the first-line imaging modality for breast cancer follow-up. To investigate the imaging characteristics and diagnostic accuracy of WBCT for incidental ovarian tumors in patients with prior breast cancer, we retrospectively reviewed a consecutive cohort of 13,845 patients with breast cancer, of whom 149 had pathologically-proven ovarian lesions. We excluded patients with ovarian diagnosis before breast cancer, CT scan not including ovary, CT-pathology interval >30 days, and severe CT artifact. Among our 60 breast cancer patients (median age, 46 years) with pathologically proven ovarian lesions, 49 patients had benign diseases, seven had primary ovarian cancer and four had ovarian metastasis from breast cancer. The histologic types of breast cancer with ovarian metastases included invasive ductal carcinoma, lobular carcinoma and angiosarcoma. Cystic ovarian lesions identified on WBCT during the breast cancer follow-up are more likely to be benign, while solid-cystic lesions are likely to be primary ovarian cancers, and solid lesions may indicate ovarian metastasis. The diagnostic accuracy, sensitivity, specificity, and areas under the receiver operating characteristic curve of WBCT were 98.3%, 100.0%, 98.0%, and 0.99 (malignant vs. benign); 90.0%, 100.0%, 85.7%, and 0.93 (metastasis vs. primary ovarian cancer), respectively. The only false positive solid lesion was a Sertoli–Leydig tumor. In conclusion, WBCT may help diagnose incidental ovarian tumors in patients with prior breast cancers and guide disease management.

## 1. Introduction

Breast cancer is the most common malignancy in women [1]. Breast cancer can metastasize to bone, lungs, liver and brain, and, although less frequently to the ovaries, either at diagnosis or recurrence [2]. The prevalence of ovarian metastases was reported to be 2.4% among young breast cancer patients [3]. Patients with prior breast cancer are also more likely to develop primary ovarian cancer [2,4,5]. Because of a high incidence of breast cancer, its metastasis to the ovaries can constitute substantially of all ovarian neoplasms [2,3]. Distinguishing ovarian metastasis from primary ovarian cancer is important, as the optimal treatments are different and correlated to prognosis. Breast cancer patients with ovarian metastasis should be treated non-surgically, whereas primary ovarian cancers still require optimal cytoreductive surgery as the mainstream treatment [4]. Before obtaining the cancer tissue, the selection of neoadjuvant chemotherapy also depends on this decision: alternative endocrine agents can be considered for the treatment of ovarian metastasis, whereas bevacizumab may be selected for primary ovarian cancer [2]. However, making this decision is also clinically challenging, because of overlapping of the patient demographics, especially when patients with prior breast cancer have an increased risk for developing primary ovarian cancer [6]. Whether any subgroup of breast cancer patients carrying ovarian tumor-like lesions might obviate oophorectomy has yet to be answered because of the increasing prevalence of pre-menopausal or peri-menopausal breast cancer patients [7].

Advanced body imaging can be offered if distant metastasis or disease recurrence is clinically suspected, and this is supported by the American Cancer Society (ACS)/American Society of Clinical Oncology (ASCO) Breast Cancer Survivorship Care [8] and the National Comprehensive Cancer Network (NCCN) Guidelines [9]. The evaluation of an ovarian lesion years after breast cancer may represent a diagnostic challenge and the possibility of a metastatic lesion should always be considered. Accumulated evidence based on ultrasonography has shown that ovarian metastases derived from breast cancers tend to be solid and vascularized [10,11,12], and bilaterality has also been reported [4,13]. With technological advancement, whole-body computed tomography (WBCT) is the first-line imaging modality for detecting distant metastasis or recurrence, with the unique capability to scan breasts and ovaries at the same time. In this regard, we conducted a more comprehensive adnexal masses morphological classification, e.g., cystic, cystic-solid and solid lesions, to exam the clinical value of routine WBCT for patients with prior breast cancer.

In this study, we aimed to investigate the imaging characteristics and diagnostic accuracy of WBCT for incidental ovarian tumors in patients with prior breast cancer.

## 2. Materials and Methods

### 2.1. Patients

The institutional review board approved this retrospective study, and informed consent was waived. We retrospectively reviewed a consecutive cohort of 13,845 patients from the breast cancer registry from 2007 to 2018, of whom 149 had pathologically-proven ovarian lesions. Exclusion criteria were (a) diagnosis date of the ovarian pathology before the breast cancer (n = 48), colon cancer (n = 2) and lung cancer (n = 1), and (b) CT scan not including ovary (n = 21), CT-pathology interval > 30 days (n = 15), and severe CT artifact (n = 2). During data curation, ovarian primary with breast metastasis (n = 3) was identified for analyzing the breast-ovarian mutual metastasis. A multidisciplinary team involving imaging experts and gynecologic oncologists discussed the work-up for asymptomatic patients with incidental pelvic lesions on WBCT (n = 40) or symptomatic patients with positive findings on transvaginal ultrasound (n = 20). Gynecologic oncologists performed pelvic examinations at three to six months and closely monitored tumor markers to decide whether the patients should be admitted for surgery. Transvaginal (n = 50) or transabdominal ultrasound (n = 5) was performed before surgery. An ultrasound-guided transvaginal biopsy was not performed in our institution. Serum tumor markers were evaluated, including the levels of carcinoma antigen (CA) 125, CA 15-3, CA19-9, and carcinoembryonic antigen (CEA).

### 2.2. WBCT

WBCT exams were carried out using multi-slice CT systems (Somatom Sensation 4 or 16, Siemens Medical Systems, Erlangen, Germany) if clinically suspected recurrence was indicated during the follow-up. Contiguous axial sections (0.5-cm thickness) were scanned in a craniocaudal direction between the lower neck and the pelvis, about 60–70 s following intravenous injection of 100 mL of iodinated contrast medium (Omnipaque 350, Amersham (Cork, Ireland)) or Optiray 350, Mallinckrodt (St. Louis, MO, USA)), at a rate of 2–3 mL/s. Routine coronal reconstruction was done for all CT studies. Two radiologists (G.L. and P.C.H.) reviewed the CT scans for all patients. Ovarian lesions were classified based on the morphology features (solid, solid-cystic or cystic) and laterality (unilateral or bilateral). The solid lesion was defined by lesions with predominant soft tissue densities with any degree of enhancement on CT. Lesions exhibiting both solid and bilateral patterns were defined as having a combined feature.

### 2.3. Histopathologic Analysis

Surgical histopathology served as the reference standard in this study according to the World Health Organization (WHO) classification 2020, reviewed by a pathologist (R.C.W.) who has 20 years of experience in gynecologic pathology. The clinical information was available for the pathological reference standard. Diagnosis was made primarily based on morphological evidence on the hematoxylin-eosin (H & E)-stained tissue sections. An additional immunohistochemical (IHC) analysis was carried out if there was any uncertainty of the diagnosis. The presence of GATA- binding protein 3 (GATA3) was suggestive of metastasis from breast cancer, whereas paired box 8 (PAX8) was suggestive of primary ovarian cancers [14]. The status of estrogen (ER), progesterone (PR) and HER2-neu receptors were used to select an appropriate adjuvant therapy.

### 2.4. Statistical Analysis

Data were analyzed using standard statistical methods on MedCalc for Windows, Version 20.008 (MedCalc Software, Ostend, Belgium). A Mann–Whitney U test was used to compare the continuous parameters, and a Fisher’s exact test was used to compare the categorical parameters, respectively. The diagnostic accuracy of WBCT in diagnosis of ovarian lesions was presented with 95% confidence intervals (CIs). The McNemar test was used to compare the accuracy, sensitivity, and specificity between groups. Areas under the receiver operating characteristic curve (AUCs) were calculated to compare diagnostic performance. A *p*-value < 0.05 indicated statistical significance in this study.

## 3. Results

### 3.1. Patient Characteristics

**Figure 1:**
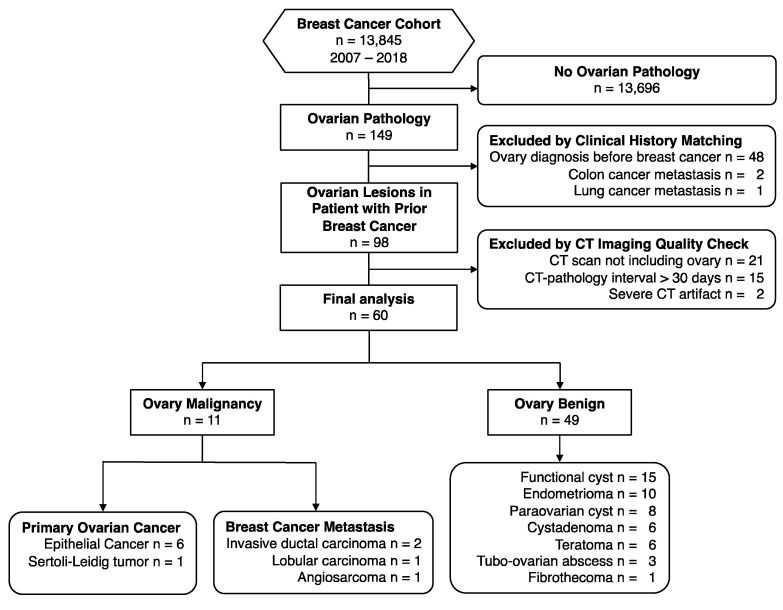
Flow diagram of the study cohort.

A total of 60 patients (median age 46 years, range 24–72) with pathological evidence of the ovary were included in the final analysis, including 49 benign and 11 malignant ovarian lesions. The flowchart of this study is demonstrated in Figure 1. The pathology of breast malignancy comprised invasive ductal carcinoma (n = 52), lobular carcinoma (n = 3), mucinous adenocarcinoma (n = 2), metastatic malignant phyllodes tumor (n = 1), papillary carcinoma (n = 1), and angiosarcoma (n = 1). The demographics of the study population are detailed in Table 1. Initial staging of the breast malignancy showed no significant differences between benign and malignant ovary groups in terms of T, N, and M stages. Ovarian pathology proved to be benign in 49 patients, including functional cysts (n = 15), endometrioma (n = 10), para-ovarian cysts (n = 8), cystadenoma (n = 6), teratoma (n = 6), tubo-ovarian abscess (n = 3), and fibrothecoma (n = 1). Prophylactic oophorectomy was offered for *BRCA* mutation carriers (n = 2) and noncarriers (n = 2), with all the pathology results yielded to be functional cysts. The benign lesions were predominantly cystic; even the fibrothecoma appeared as a cystic mass lesion in this cohort. The malignant lesions were solid or solid-cystic. Patients with malignant ovarian lesions had a shorter diagnosis interval (8 vs. 29 months, *p* = 0.044), and were associated with the M1 stage at diagnosis (*p* = 0.003), as compared with patients with benign ovarian lesions.

### 3.2. Malignant Ovarian Lesions

**Figure 2:**
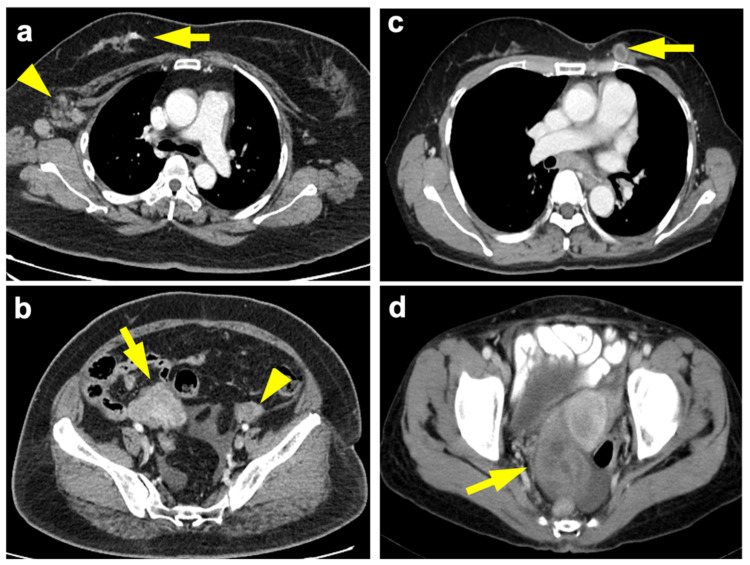
(**a**) 54-year-old woman, breast invasive lobular carcinoma (T3N2aM1) with ovarian metastasis. Contrast-enhanced axial chest CT shows infiltrative tumor at the upper outer and upper inner quadrant of the right breast (arrow) with right axillary nodal metastases (arrowhead). (**b**) Contrast-enhanced axial pelvic CT done at the same time showed a 5.9-cm solid tumor involving the right ovary (arrow) and a normal left ovary (arrowhead), based on final pathology. Immunohistochemistry of the breast tumor exhibited ER+, PR+ and HER2−. The levels of CA125, CA15-3, CA19-9 and CEA at documented metastasis were 38.4 U/mL, 15.4 U/mL, 21.4 U/mL, and 0.84 ng/mL, respectively. (**c**) 55-year-old woman, ovarian serous adenocarcinoma (T3cN1M1) with breast metastasis. Contrast-enhanced axial chest CT shows necrotic tumors at upper inner quadrant of the left breast (arrow). (**d**) Contrast-enhanced axial pelvic CT 27 months ago showed complex cystic tumors (right 6.0 cm, left 2.2 cm) involving the ovaries (arrow). Immunohistochemistry of the breast tumor exhibited ER−, PR+, HER2+. The levels of CA125, CA15-3, CA19-9, and CEA at documented metastasis were 906.7 U/mL, 161.8 U/mL, 65.18 U/mL, 1.3 ng/mL, respectively. Recurrence was found eight months after diagnosis of breast metastasis and patient died in 33 months.

The 11 malignant ovarian lesions comprised primary ovarian epithelial cancer (n = 6), moderately differentiated Sertoli–Leydig cell tumor (n = 1), and metastasis from breast cancer (n = 4). Ovarian metastases from breast cancer originated from invasive ductal carcinoma (n = 2), lobular carcinoma (n = 1, Figure 2a,b) and angiosarcoma (n = 1). Their clinicopathological features are detailed in Table 2. 

Ovarian lesions were detected as the first extra-mammary presentations for all the 11 patients, with one breast cancer metastasis and five primary ovarian malignancies being the only extra-mammary solid organ metastasis. Four of them were symptomatic (pain, dysmenorrhea, urinary frequency), and the pelvic ultrasound findings prompted the WBCT examination. The other seven patients were asymptomatic, with the ovarian lesions identified incidentally on routine WBCT examination. Primary ovarian cancer (n = 7) occurred more commonly than breast cancer with ovarian metastasis (n = 4), with a ratio of 1.75. No statistically significant differences were found between the ovarian primary versus metastasis in terms of the sizes of ovarian tumors (median [range], 8.4 cm [4.9–25.2] vs. 8.6 cm [4.7–13.7], *p* = 1.000) or the sizes of their original breast tumors (2.2 cm [1.2–7.4] vs. 5.1 cm [1.5–9.3], *p* = 0.130). Primary ovarian cancer tended to show mixed solid-cystic, whereas the breast cancer with ovarian metastasis showed a solid appearance (*p* = 0.015) excepting the primary ovarian Sertoli–Leydig cell tumor being solid. Bilateral ovarian lesions were found in two out of the four patients with ovarian metastasis (50%). The bilaterality of the ovarian lesions, however, did not demonstrate a statistically significant difference between primary ovarian cancers and metastasis from breast cancer. The interval of ovarian metastasis was shorter than the primary ovarian cancer (median four vs. 10 months), albeit not statistically significant. No statistical significance was observed between the ovarian metastasis and primary ovarian cancer patients in terms of their serum levels of CA 125, CA 15-3, CA19-9, and CEA. Notably, the elevation of CEA or CA19-9 or distant metastasis to the bone was only observed in breast cancer with ovarian metastasis but not in primary ovarian malignancies. The tissue expressions of estrogen receptor (ER), progesterone receptor (PR), Her2 = human epidermal growth factor receptor 2 (HER2), were found in all three breast cancers with ovarian metastases.

### 3.3. Diagnostic Performance

The diagnostic accuracy, sensitivity, and specificity, and areas under the receiver operating characteristic curve of WBCT were 98.3%, 100.0%, 98.0%, and 0.99 (malignant vs. benign); 90.0%, 100.0%, 85.7%, and 0.93 (metastasis vs. primary ovarian cancer), respectively. The cross-tabulation of the results is detailed in Table 3. The solid feature had a significantly higher area under the receiver operating characteristic curve, as compared with the bilaterality feature (0.99 vs. 0.59, *p* < 0.0001) or the combined feature (0.99 vs. 0.64, *p* < 0.0001). The only false positive solid lesion was a Sertoli–Leydig tumor.

### 3.4. Breast-Ovarian Mutual Metastasis

We identified an interesting radiological phenotype of breast primary with ovarian metastasis (n = 4) or ovarian primary with breast metastasis (n = 3, Figure 2c,d), defined as the mutual metastasis group in this study, as opposed to patients with double primary malignancies (n = 7). Mutual metastasis patients exhibited more solid rather than solid-cystic ovarian lesions (*p* = 0.029), and more distant metastatic lesions (*p* = 0.010), as compared with double primary cancer patients. Their age, diagnostic interval, as well as tissue expression of ER, PR, and HER2 were not statistically significant.

## 4. Discussion

We found that cystic ovarian lesions identified on WBCT during the breast cancer follow-up were more common to be benign, while solid-cystic lesions were likely to be primary ovarian cancers, and solid lesions possibly to be ovarian metastasis. Metastases to the ovaries from breast cancer were reported to be usually bilateral [2,15,16]. We found that the solid feature, either uni- or bilateral, was the most indicative for ovarian metastasis based on the histopathological evidence in the present study. Through the subgroup analysis, we identified an interesting subset phenotype of ovarian cancer, i.e., breast cancer metastasizing to ovaries or ovarian cancer metastasizing to breasts, that exhibited predominantly solid ovarian tumors, as opposed to the solid-cystic pattern in patients with double primary cancers originating from both breasts and ovaries. The present study suggested WBCT may not only detect malignant ovarian lesions but also differentiate breast metastases from primary ovarian malignancies.

Clinicopathologic characteristics of primary breast cancer provides the initial information to select patients having an increased risk of ovarian metastasis. Lobular carcinoma and invasive ductal carcinoma are the dominant pathological types with ovarian metastasis in our study. Invasive ductal carcinoma makes up the majority of cases, as shown in our cohort. Lobular carcinoma comprises only 4–15% of all malignant neoplasms of the breast [4,7,15,17,18,19] but has three times greater metastatic tendency occurring in ovaries [17,18]. About 19% of lobular carcinoma with distant metastasis also have lesions in the ovary or uterus [19]. Therefore, in a patient with a history of lobular carcinoma, a new malignant ovarian mass could be more suspicious for metastatic disease. The differential diagnosis includes sex cord-stromal tumors, lymphoma/leukemia, and desmoplastic small round cell tumor of the ovary [20]. In line with our study, the majority of breast cancers with ovarian metastases were reported to have tissue expression of ER, PR, and HER2 [21]. It has been reported that breast cancer metastasis to the ovaries usually occurs in the younger, premenopausal population [7,22]; however, in our cohort, patients’ age did not differ, and was mainly at perimenopausal age, as supported by other studies [4,21]. The time interval between the diagnosis of initial breast cancer with succeeding primary ovarian cancer was shorter than with ovarian metastasis [23].

Understanding the incidence is helpful in this clinical scenario. The probability of ovarian metastasis in breast cancer patients is estimated to be 6.7% (4/60) in the present study, and ovarian metastases are found mostly in women with advanced stages of breast cancer. Based on the present study, a newly found adnexal mass in a woman with prior breast cancer is more commonly benign. The benign and malignant disease resulted in a 4.5:1 ratio, which is in line with two independent cohort studies with 129 patients [5] and 54 patients [24], respectively. Within the scope of malignant masses, we demonstrated that the primary ovarian cancer and metastatic disease resulted in a 1.75:1 ratio. This is supported by another study with 27 patients diagnosed with primary epithelial ovarian cancer and nine had metastatic disease, resulting in a 3:1 ratio [4].

The finding of ovarian metastases in the ovaries when the disease has the systemic character requires systemic treatment and pelvic/abdominal surgery to remove the adnexal mass is not always required. On the other hand, even the incidental finding of a small solid-cystic or purely solid adnexal mass on the WBCT or pelvic transvaginal sonography in a woman with a history of breast cancer makes an early differential diagnosis extremely important. Our result is supported by the literature, showing even small primary tumors may result in metastatic disease to the ovaries [21,24]. Metastases to the ovaries from breast cancer can be relatively small solid masses; in nearly half of cases the size of the ovary is not enlarged [15,25]. From hematogenous spread, the ovarian metastases from breast cancer are mainly nested in the ovarian medulla and/or cortex [26,27]. In contrast, primary ovarian cancers are more commonly located in the ovarian surface epithelium and superficial cortex, accompanied by fallopian tube involvement [28]. In line with our study, the transvaginal ultrasonography features of ovarian metastases appear to be solid if involved by lymphoma or metastases from the stomach, breast and uterus, whereas being multicystic with irregular borders if derived from the colon, rectum or biliary tract [10,11,12]. Unilateral tumors appear more often in the primary ovarian cancer group and bilateral disease in the ovarian metastasis [4,13]. Our study showed the laterality added little value to select breast cancer metastasis in the present cohort.

The breast-ovarian mutual metastasis patients also demonstrated more extensive distant metastasis at the time of ovarian diagnosis. Other poor prognostic factors of breast cancer, such as co-existent other metastatic sites [3], stage III–IV [24,29], and large positive lymph nodes [19], are positively correlated with metastatic breast cancer to the ovaries. Although *BRCA* mutations increase the risk of ovarian cancer, the incidence of ovarian metastasis did not differ between the *BRCA* mutation carriers and noncarrier breast cancer patients [30]. It has been reported that 75% of patients were asymptomatic, and 42% of patients exhibited advanced-stage pelvic extent or extra-abdominal metastases [7], highlighting the importance of WBCT in detecting distant metastasis. A study based on PET-CT also supported that breast cancer with solid ovarian metastasis with multiple metastases included omentum, liver, and bone [31], whereas synchronous double primaries demonstrated bilateral adnexal cystic tumors with omentum and peritoneal spread [31]. Because both cancer and the physiological change of the ovary might demonstrate avid glucose uptake, the morphological features from the CT part of PET-CT might also aid the differential diagnosis in this regard.

### Limitation

Some limitations warrant notification while interpreting our data. First, this is a single-center retrospective study. Due to the limited number of patients with malignant cases, subgroup analysis of metastasis type and imaging features of malignant ovaries could not be carried out simultaneously. That could introduce bias to the data, and the sample size was not sufficient to uncover modest differences. To prevent selection bias and uncontrolled confounding factors, such as non-surgical chemotherapy regimens, we did not intend to investigate the impact on prognosis, but focused on the utility of WBCT in the clinical decisions pathway. Nevertheless, to our knowledge, the present study includes the largest number of ovarian cancers for WBCT analysis. Second, the case number in the ovarian malignancy group might be underestimated, because our study defined the ground truth to be histopathological evidence only. The prevalence of perimenopausal breast cancer patients with ovarian metastasis might lead the readers to think that bilateral oophorectomy might be beneficial even when the contralateral ovary appears to be normal [7]. The number of cases with malignant ovarian lesions in the present report is very small, so one must be very cautious with the interpretation of the results. The current results translate to select the surgical candidate and improve survival, but might warrant a larger series to confirm. 

## 5. Conclusions

In conclusion, cystic ovarian lesions identified on WBCT during the breast cancer follow-up are more common to be benign, while solid-cystic lesions are likely to be primary ovarian cancers, and solid lesions possibly to be ovarian metastasis. WBCT may help diagnose incidental ovarian tumors on WBCT in patients with prior breast cancer and guide the disease management.

## Figures and Tables

**Table 1:** Demographics of patients with prior breast cancer having incidental ovarian tumors.

	All	Ovary Malignancy	Ovary Benign	*p*-Value
	(n = 60)	(n = 11)	(n = 49)	
Age, median (year)	46 (24, 72)	51 (32, 60)	45 (24, 72)	0.203
Breast pathology				0.390
Invasive ductal carcinoma	52	9 (81.8%)	43 (87.8%)	
Lobular carcinoma	3	1 (9.1%)	2 (4.1%)	
Mucinous adenocarcinoma	2	0 (0.0%)	2 (4.1%)	
Malignant phyllodes tumor	1	0 (0.0%)	1 (2.0%)	
Papillary carcinoma	1	0 (0.0%)	1 (2.0%)	
Angiosarcoma	1	1 (9.1%)	0 (0.0%)	
Morphology				0.000
Solid or solid-cystic	11	11 (100.0%)	1 (2.0%)	
Cystic	49	0 (0.0%)	48 (98.0%)	
Laterality				0.154
Bilateral	8	3 (27.3%)	5 (10.2%)	
Unilateral	52	8 (72.7%)	44 (89.8%)	
Interval, median (month)	27 (0, 140)	8 (0, 42)	29 (1, 140)	0.044
T stage				0.345
3–4	9	3 (27.3%)	6 (12.2%)	
1–2	51	8 (72.7%)	43 (87.8%)	
N stage				1.000
123	24	4 (36.4%)	20 (40.8%)	
0	36	7 (63.6%)	29 (59.2%)	
M stage				0.003
1	8	5 (45.5%)	3 (6.1%)	
0	52	6 (54.5%)	46 (93.9%)	

Note—Numbers in parentheses are ranges or percentage. *p*-value based on Mann-Whitney test/Fisher Exact.

**Table 2:** Clinicopathological features of the malignant ovarian lesions.

ID	Origin	Breast	Age	TNM	Interval (m)	Ovary	Modality	Feature *	Lat ^†^	Only	CA125	CA15-3	CA19-9	CEA
1	Breast	IDC	48	T4N1M1	6	mIDC	CT	S	B	N	+	+	−	+
2	Breast	IDC	55	T2NM1	0	mIDC	CT	S	U	Y	−	−	+	−
3	Breast	ILC	54	T3N2aM1	1	mILC	CT	S	U	N	+	−	−	−
4	Breast	AS	40	T2N0M1	32	mAS	US	S	B	N	−	−	N/A	−
5	Ovary	IDC	58	T1cN0M0	42	SC	US	SC	B	Y	+	−	−	−
6	Ovary	IDC	44	T2N0M0	8	SC	US	SC	U	Y	−	−	−	−
7	Ovary	IDC	51	T1cN3aM0	34	CCC	US	SC	U	N	−	−	N/A	−
8	Ovary	IDC	32	T2N0M0	10	CCC	CT	SC	U	Y	−	−	N/A	−
9	Ovary	IDC	55	T1cN2aM0	0	SC	CT	SC	U	Y	+	+	−	N/A
10	Ovary	IDC	60	T1N0M0	1	SC	CT	SC	U	Y	−	−	−	−
11	Ovary	IDC	38	T4N3M1	41	SL	CT	S	U	N	N/A	N/A	N/A	N/A

Note—AS, angiosarcoma; CCC, clear cell carcinoma; IDC, invasive ductal carcinoma; ILC, invasive lobular carcinoma; MC, mucinous carcinoma; SC, serous carcinoma; UC, undifferentiated carcinoma; mIDC, metastatic invasive ductal carcinoma; mAS, metastatic angiosarcoma; mILC, metastatic lobular carcinoma; CT, computed tomography; US, ultrasonography; †, of ovary malignancy. CT features: S, solid; SC, solid-cystic; Lat ^†^, laterality for ovarian lesions: B; bilateral; U, unilateral. Only, ovary as the only extra-mammary solid organ metastasis. +, tumor markers elevated; −, tumor makers within normal range; N/A, non-available. TNM stages for breast cancer; Angiosarcoma staging based on staging for soft tissue sarcoma of the trunk and extremities. *, metastasis from breast cancer vs primary ovarian cancer, *p* < 0.05.

**Table 3:** Diagnostic accuracy of whole-body computed tomography for ovarian lesions.

	FN	TP	TN	FP	Accuracy	Sensitivity	Specificity	PPV	NPV
Malignant vs. Benign									
Solid/SC	0	11	48	1	98.3 (91.1–100.0)	100.0 (71.5–100.0)	98.0 (89.1–99.9)	91.7 (61.5–99.8)	100.0 (92.6–100.0)
Bilateral	8	3	44	5	78.3 (65.8–87.9)	27.3 (6.0–61.0)	89.8 (77.8–96.6)	37.5 (8.5–75.5)	84.6 (71.9–93.1)
Combined	8	3	49	0	86.7 (75.4–94.1)	27.3 (6.0–61.0)	100.0 (92.7–100.0)	100.0 (29.2–100.0)	86.0 (74.2–93.7)
Metastasis vs. Primary									
Solid	0	4	6	1	90.9 (58.7–99.8)	100.0 (39.8–100.0)	85.7 (42.1–99.6)	80.0 (28.4–99.5)	100.0 (54.1–100.0)
Bilateral	2	2	6	1	72.7 (39.0–94.0)	50.0 (6.8–93.2)	85.7 (42.1–99.6)	66.7 (9.4–99.2)	75.0 (34.9–96.8)
Combined	2	2	7	0	81.8 (48.2–97.7)	50.0 (6.8–93.2)	100.0 (59.0–100.0)	100.0 (15.8–100.0)	77.8 (40.0–97.2)

Note—Data are numbers. In parentheses are 95% confidence intervals. SC, solid-cystic. AUC = areas under the receiver operating characteristics curve, TP = true positive, TN = true negative, FP = false positive, FN = false negative.

## Data Availability

All data generated or analyzed during this study are included in this published article.
